# Development and validation of nomograms to recurrence and survival in patients with early-stage cervical adenocarcinoma

**DOI:** 10.1007/s00432-023-05068-4

**Published:** 2023-08-01

**Authors:** Xintao Wang, Wenpei Shi, Xiaowen Pu, Yan Hu, Ruiying Chen, Haiyan Zhu

**Affiliations:** 1 Department of Gynecology, Shanghai First Maternity and Infant Hospital, School of Medicine, Tongji University, Shanghai, China; 2 Clinical Research Unit, Shanghai First Maternity and Infant Hospital, School of Medicine, Tongji University, Shanghai, China; 3 Department of Gynecology, The First Affiliated Hospital of Wenzhou Medical University, Wenzhou, China

**Keywords:** Nomogram, Recurrence, Survival, Cervical adenocarcinoma

## Abstract

**Purpose:**

Cervical adenocarcinoma is one of the most common types of cervical cancer and its incidence is increasing. The biological behavior and treatment outcomes of cervical adenocarcinoma (CA) differ from those of squamous cell carcinoma (SCC). We sought to develop a model to predict recurrence and cancer-specific survival (CSS) deaths in CA patients.

**Methods:**

131 patients were included in model development and internal validation, and patients from the SEER database (*N* = 1679) were used for external validation. Multivariable Cox proportional hazards regression analysis was used to select predictors of relapse-free survival (RFS) and CSS and to construct the model, which was presented as two nomograms. Internal validation of the nomograms was performed using the bootstrap resampling method.

**Results:**

Age, FIGO (International Federation of Gynecology and Obstetrics) stage, size of the tumor, lymph metastasis and depth of invasion were identified as independent prognostic factors for RFS, while age, FIGO stage, size of the tumor and number of positive LNs were identified as independent prognostic factors for CSS. The nomogram of the recurrence model predicted 2- and 5-year RFS, with optimism adjusted c-statistic of 75.41% and 74.49%. Another nomogram predicted the 2- and 5-year CSS with an optimism-adjusted c-statistic of 83.22% and 83.31% after internal validation; and 68.6% and 71.33% after external validation.

**Conclusions:**

We developed and validated two effective nomograms based on static nomograms or online calculators that can help clinicians quantify the risk of relapse and death for patients with early-stage CA.

**Supplementary Information:**

The online version contains supplementary material available at 10.1007/s00432-023-05068-4.

## Introduction

Cervical cancer is the fourth most commonly diagnosed cancer among women worldwide, with approximately 604,000 new cases and 342,000 deaths in 2020 (Ward et al. 2020). Over the past few decades, the incidence and mortality rates of cervical cancer have steadily declined with the development of cervical cancer screening and HPV vaccines in high-income countries (Siegel et al. 2021). However, invasive adenocarcinoma, the second most common histologic type of invasive cervical carcinoma, has shown an increasing trend in incidence over the past two decades (Islami et al. 2019). Therefore, more attention should be paid to the prevention and treatment of cervical adenocarcinoma. Increasing evidence has shown that the genomic alterations, biological behavior, treatment outcomes, and prognostic factors of cervical adenocarcinoma (CA) differ from those of squamous cell carcinoma (SCC) (Ni et al. 2021). More recently, Levinson et al. reported that tumor size was the highest risk factor for recurrence of cervical adenocarcinoma, while the depth of invasion was the highest risk factor for recurrence of squamous cell carcinoma (Bhatla et al. 2019). Therefore, it is necessary to explore the prognostic factors of CA and develop a predictive model for predicting the prognosis of CA and optimizing treatment strategies. In this study, we hoped to develop the nomograms for relapse-free survival (RFS) and cancer-specific survival (CSS) using our central database as well as the Surveillance, Epidemiology, and End Results (SEER) database to more accurately evaluate the prognosis of patients with cervical adenocarcinoma.

## Methods

### Patient data

All consecutive patients diagnosed with cervical adenocarcinoma (CA) at the First Affiliated Hospital of Wenzhou Medical University, China between December 2008 and September 2018 were eligible for this study. This study complied with the Declaration of Helsinki. In the study, patients had signed written informed consents to be included. This research followed the ethical principles of the First Affiliated Hospital of Wenzhou Medical University. Clinical and pathologic information were obtained from patient files and pathology reports. Only patients with stage I–II (FIGO 2009) CA were included.

### Predictors

The following predictors were selected for model development: age, grade, FIGO 2009 stage, surgery manner (laparotomy or laparoscopy), tumor size, differentiation (low, medium, and high differentiation), lymph metastasis (yes or no), number of positive lymph nodes (LNs), lymph-vascular space invasion (LVSI) (yes or no), infiltration depth, resection margin (positive or negative), radiation (yes or no), chemotherapy (yes or no), D-Dimer, platelet, total cholesterol (TC), triglyceride (TG), high-density lipoprotein (HDL), low-density lipoprotein (LDL), glucose (GLU), hemoglobin (HB), red blood cell (RBC), (the above blood tests were completed within 1 week before the operation), red blood cell after the operation (RBC after) and hemoglobin after the operation (HB after). Both RBC after and HB after were tested the next day after the operation).

### Handling of missing data

In the study, there were 146 cases in the original database, of which 15 cases were lost to follow-up, giving a total of 131 cases were included in the analysis. Missing values, including both categorical and continuous variables, were imputed using random forest method for five times. The model results of different imputed datasets were combined according to Rubin’s rules, and the pooled C-index value and 95%CI were also calculated using mice:pool function.

### Transformation of the predictors

To facilitate the model’s use and interpretation in practice, continuity variables such as age and tumor size were transformed into categorical variables. The optimal cutoff point of age was selected using the log-rank tests. According to the log-rank test, age at diagnosis was categorized as: ≤ 55, > 55 years. According to the FIGO staging system, tumor size (defined as the maximum measurement of horizontal diffusion or surface diameter in the ultrasound field) was divided into two groups: ≤ 40 and > 40 mm.

### Predictor selection

The primary endpoint focused in this study is patient recurrence. Univariate Cox regression analysis was used to determine the prognostic factors associated with the total recurrence rate and a *p* value < 0.05 was considered statistically significant. Hence, eight variables were ascertained in subsequent analysis: age, stage, tumor size, lymph metastasis, number of positive LNs, infiltration depth, edge positive, and radiation. Considering the results of univariate Cox analysis, the clinical relevance of the variables, and the sample size, multiple multifactor models were established. The model with the highest C-index were selected as the final prediction model. Consequently, five variables, including age, stage, size of the tumor, lymph metastasis and depth of invasion, were included in the final model.

For the survival model, the study endpoint of this study was death specifically attributed to CA. Survival time was calculated from the time of diagnosis until death attributed to CA or last follow-up. Through the same statistical method as above, four variables, including age, stage, tumor size and number of positive LNs, were included in the survival model.

### Model development and internal validation

To visualize the predictive models, two nomograms for predicting the 2- and 5-year relapse-free survival (RFS) and cancer-specific survival (CSS) in patients were further constructed. Then developed nomograms were internally validated and calibrated using the bootstrap resampling (*B* = 1000) approach as assessed by the C-index and calibration curves. The survival prediction model was validated in SEER database externally.

### SEER data extraction and external validation of survival model

Cases with CA were identified using the International Classification of Diseases for Oncology, third edition (ICD-O-3). Histology code: 8140/3, 8144/3, 8147/3, 8200/3, 8210/3, 8241/3, 8244/3, 8255/3, 8260–8263/3, 8310/3, 8313/3, 8323/3, 8380/3, 8382/3, 8384/3, 8430/3, 8441/3, 8460/3, 8461/3, 8480–8482/3, and 8490/3 (Fritz et al. 2000; Lu and Chen 2014). We collected data on confirmed CA cases from the SEER registry (*n* = 1679) from 2004 to 2015 for external validation. The seven variables collected from the database were age, stage, tumor size, number of positive LNs, survival time, cause of death, and vital status. Patients diagnosed with stage III or IV disease, no follow-up data, no lymph node examination results, and missing values of modeling variables were excluded.

### Statistical analysis

Continuous variables are described as mean ± standard deviation (SD) or median with interquartile range (IQR) values, depending on whether they are normal or non-normal. Categorical variables are shown as numbers and percentages for each group. Cox proportional hazards regression analysis was used to construct predictive models that were presented as static nomograms and dynamic web-based nomograms. The nomogram for the recurrence model was internally validated with a bootstrap resampling method. The prediction performance of the survival nomogram was assessed by resampling techniques for internal validation and on the external validation cohort from SEER database. All statistical analyses were performed using R software (version 3.6.3). *p* < 0.05 was considered statistically significant.

## Results

### Clinical characteristics

Excluding 15 patients who lost follow-up, a total of 131 patients diagnosed with CA at the First Affiliated Hospital of Wenzhou Medical University between December 2008 and September 2018 were enrolled. The patients’ baseline characteristics are shown in Tables 1 and 2. The mean age of the entire cohort was 49.8 ± 10.1. Of the patients, 95 (72.5%) cases, and 36 (27.5%) cases were ≤ 55, and > 55 years old, respectively; 80 (61.1%) cases and 15 (11.5%) cases had tumor sizes ≤ 40, and > 40 mm, respectively. The number of patients with stage I and stage II were 97 (74%) and 34 (26%). The number of patients with or without lymph metastasis was 22 (16.8%) and 109 (83.2%); the depth of invasion of ≤ 2/3 and > 2/3 were 81 (61.8%), and 49 (37.4%), respectively.

**Table 1:** Clinical characteristics of the recurrence group and the nonrecurrent group from raw data

Characteristics	No-reverse	Reverse	Overall	*p*
	(*N* = 112)	(*N* = 19)	(*N* = 131)	
Age(years)				0.068
≤ 55	85 (75.9%)	10 (52.6%)	95 (72.5%)	
> 55	27 (24.1%)	9 (47.4%)	36 (27.5%)	
Stages				0.01
I	88 (78.6%)	9 (47.4%)	97 (74.0%)	
II	24 (21.4%)	10 (52.6%)	34 (26.0%)	
Surgery manner				0.892
Laparotomy	99 (88.4%)	16 (84.2%)	115 (87.8%)	
Laparoscopy	13 (11.6%)	3 (15.8%)	16 (12.2%)	
Tumour size (mm)				0.001
≤ 40	73 (65.2%)	7 (36.8%)	80 (61.1%)	
> 40	8 (7.1%)	7 (36.8%)	15 (11.5%)	
Missing	31 (27.7%)	5 (26.3%)	36 (27.5%)	
Differentiation				0.295
Well differentiated	22 (19.6%)	2 (10.5%)	24 (18.3%)	
Moderate differentiated	44 (39.3%)	8 (42.1%)	52 (39.7%)	
Poorly differentiated	26 (23.2%)	8 (42.1%)	34 (26.0%)	
Missing	20 (17.9%)	1 (5.3%)	21 (16.0%)	
Lymph metastasis				< 0.001
No	99 (88.4%)	10 (52.6%)	109 (83.2%)	
Yes	13 (11.6%)	9 (47.4%)	22 (16.8%)	
Number of positive LNs				< 0.001
Median [Min, Max]	0 [0, 6.00]	0 [0, 13.0]	0 [0, 13.0]	
Vascular invasion				0.252
No	93 (83.0%)	13 (68.4%)	106 (80.9%)	
Yes	15 (13.4%)	5 (26.3%)	20 (15.3%)	
Missing	4 (3.6%)	1 (5.3%)	5 (3.8%)	
Infiltration depth				0.006
≤ 2/3	75 (67.0%)	6 (31.6%)	81 (61.8%)	
> 2/3	36 (32.1%)	13 (68.4%)	49 (37.4%)	
Missing	1 (0.9%)	0 (0%)	1 (0.8%)	
Resection margin				0.067
Negative	111 (99.1%)	16 (84.2%)	127 (96.9%)	
Positive	1 (0.9%)	2 (10.5%)	3 (2.3%)	
Missing	0 (0%)	1 (5.3%)	1 (0.8%)	
Radiation				0.087
No	74 (66.1%)	8 (42.1%)	82 (62.6%)	
Yes	21 (18.8%)	7 (36.8%)	28 (21.4%)	
Missing	17 (15.2%)	4 (21.1%)	21 (16.0%)	
Chemotherapy				0.681
No	60 (53.6%)	9 (47.4%)	69 (52.7%)	
Yes	48 (42.9%)	10 (52.6%)	58 (44.3%)	
Missing	4 (3.6%)	0 (0%)	4 (3.1%)	
d-Dimer (mg/L)				0.701
Mean (SD)	2.07 (20.5)	0.259 (0.446)	1.81 (18.9)	
Platelet (10^9^/L)				0.295
Mean (SD)	251 (77.2)	231 (60.8)	248 (75.2)	
Missing	1 (0.9%)	1 (5.3%)	2 (1.5%)	
TC (mmol/L)				0.168
Mean (SD)	5.28 (1.33)	4.79 (0.804)	5.22 (1.29)	
Missing	10 (8.9%)	4 (21.1%)	14 (10.7%)	
TAG (mmol/L)				0.703
Mean (SD)	15.4 (137)	1.82 (1.58)	13.6 (128)	
Missing	10 (8.9%)	4 (21.1%)	14 (10.7%)	
HDL (mmol/L)				0.456
Mean (SD)	1.37 (0.363)	1.45 (0.432)	1.38 (0.372)	
Missing	10 (8.9%)	4 (21.1%)	14 (10.7%)	
LDL (mmol/L)				0.134
Mean (SD)	3.10 (1.21)	2.61 (0.741)	3.03 (1.17)	
Missing	10 (8.9%)	4 (21.1%)	14 (10.7%)	
GLU (mmol/L)				0.833
Mean (SD)	7.21 (11.3)	6.64 (2.53)	7.12 (10.5)	
Missing	6 (5.4%)	1 (5.3%)	7 (5.3%)	
HB (g/L)				0.754
Mean (SD)	125 (16.5)	127 (19.8)	125 (16.9)	
Missing	0 (0%)	1 (5.3%)	1 (0.8%)	
HB after (g/L)				0.974
Mean (SD)	109 (89.3)	108 (12.7)	109 (83.5)	
Missing	3 (2.7%)	3 (15.8%)	6 (4.6%)	
RBC (10^12^/L)				0.222
Mean (SD)	4.29 (0.479)	4.14 (0.411)	4.27 (0.471)	
Missing	0 (0%)	1 (5.3%)	1 (0.8%)	
RBC after (10^12^/L)				0.397
Mean (SD)	3.47 (0.562)	3.59 (0.534)	3.49 (0.558)	
Missing	1 (0.9%)	1 (5.3%)	2 (1.5%)	

*LNs* lymph nodes

**Table 2:** Clinical characteristics of the survival group and the dead group from raw data

Characteristics	Survive	Dead	Overall	*p*
	(*N* = 118)	(*N* = 13)	(*N* = 131)	
Age (years)				0.055
≤ 55	89 (75.4%)	6 (46.2%)	95 (72.5%)	
> 55	29 (24.6%)	7 (53.8%)	36 (27.5%)	
Stages				0.037
I	91 (77.1%)	6 (46.2%)	97 (74.0%)	
II	27 (22.9%)	7 (53.8%)	34 (26.0%)	
Surgery manner				0.938
Laparotomy	103 (87.3%)	12 (92.3%)	115 (87.8%)	
Laparoscopy	15 (12.7%)	1 (7.7%)	16 (12.2%)	
Tumour size (mm)				0.003
≤ 40	76 (64.4%)	4 (30.8%)	80 (61.1%)	
> 40	10 (8.5%)	5 (38.5%)	15 (11.5%)	
Missing	32 (27.1%)	4 (30.8%)	36 (27.5%)	
Differentiation				0.724
Well differentiated	22 (18.6%)	2 (15.4%)	24 (18.3%)	
Moderate differentiated	47 (39.8%)	5 (38.5%)	52 (39.7%)	
Poorly differentiated	29 (24.6%)	5 (38.5%)	34 (26.0%)	
Missing	20 (16.9%)	1 (7.7%)	21 (16.0%)	
Lymph metastasis				0.001
No	103 (87.3%)	6 (46.2%)	109 (83.2%)	
Yes	15 (12.7%)	7 (53.8%)	22 (16.8%)	
Number of positive LNs				< 0.001
Median [Min, Max]	0 [0, 11.0]	2.00 [0, 13.0]	0 [0, 13.0]	
Vascular invasion				0.621
No	97 (82.2%)	9 (69.2%)	106 (80.9%)	
Yes	17 (14.4%)	3 (23.1%)	20 (15.3%)	
Missing	4 (3.4%)	1 (7.7%)	5 (3.8%)	
Infiltration depth				0.03
≤ 2/3	77 (65.3%)	4 (30.8%)	81 (61.8%)	
> 2/3	40 (33.9%)	9 (69.2%)	49 (37.4%)	
Missing	1 (0.8%)	0 (0%)	1 (0.8%)	
Resection margin				0.653
Negative	116 (98.3%)	11 (84.6%)	127 (96.9%)	
Positive	2 (1.7%)	1 (7.7%)	3 (2.3%)	
Missing	0 (0%)	1 (7.7%)	1 (0.8%)	
Radiation				0.215
No	76 (64.4%)	6 (46.2%)	82 (62.6%)	
Yes	23 (19.5%)	5 (38.5%)	28 (21.4%)	
Missing	19 (16.1%)	2 (15.4%)	21 (16.0%)	
Chemotherapy				0.358
No	64 (54.2%)	5 (38.5%)	69 (52.7%)	
Yes	50 (42.4%)	8 (61.5%)	58 (44.3%)	
Missing	4 (3.4%)	0 (0%)	4 (3.1%)	
d-Dimer (mg/L)				0.729
Mean (SD)	0.162 (0.317)	0.0754 (0.215)	0.154 (0.308)	
Platelet (10^9^/L)				0.452
Mean (SD)	252 (73.1)	233 (61.0)	250 (72.1)	
Missing	1 (0.8%)	1 (7.7%)	2 (1.5%)	
TC (mmol/L)				0.152
Mean (SD)	5.30 (1.26)	4.69 (0.885)	5.24 (1.24)	
Missing	12 (10.2%)	2 (15.4%)	14 (10.7%)	
TAG (mmol/L)				0.743
Mean (SD)	14.9 (134)	1.53 (0.941)	13.7 (128)	
Missing	12 (10.2%)	2 (15.4%)	14 (10.7%)	
HDL (mmol/L)				0.523
Mean (SD)	1.36 (0.344)	1.45 (0.424)	1.37 (0.351)	
Missing	12 (10.2%)	2 (15.4%)	14 (10.7%)	
LDL (mmol/L)				0.263
Mean (SD)	3.02 (1.11)	2.66 (0.751)	2.99 (1.09)	
Missing	12 (10.2%)	2 (15.4%)	14 (10.7%)	
GLU (mmol/L)				0.998
Mean (SD)	6.10 (1.81)	7.12 (2.84)	6.21 (1.95)	
Missing	7 (5.9%)	0 (0%)	7 (5.3%)	
HB (g/L)				0.839
Mean (SD)	126 (16.2)	132 (16.5)	126 (16.3)	
Missing	1 (0.8%)	0 (0%)	1 (0.8%)	
HB after (g/L)				0.921
Mean (SD)	110 (87.1)	106 (13.3)	109 (82.9)	
Missing	5 (4.2%)	1 (7.7%)	6 (4.6%)	
RBC (10^12^/L)				0.076
Mean (SD)	4.29 (0.466)	4.05 (0.435)	4.27 (0.467)	
Missing	1 (0.8%)	0 (0%)	1 (0.8%)	
RBC after (10^12^/L)				0.562
Mean (SD)	3.48 (0.553)	3.58 (0.590)	3.49 (0.555)	
Missing	1 (0.8%)	0 (0%)	1 (0.8%)	

### Cox regression analysis of disease recurrence

**Fig. 1:**
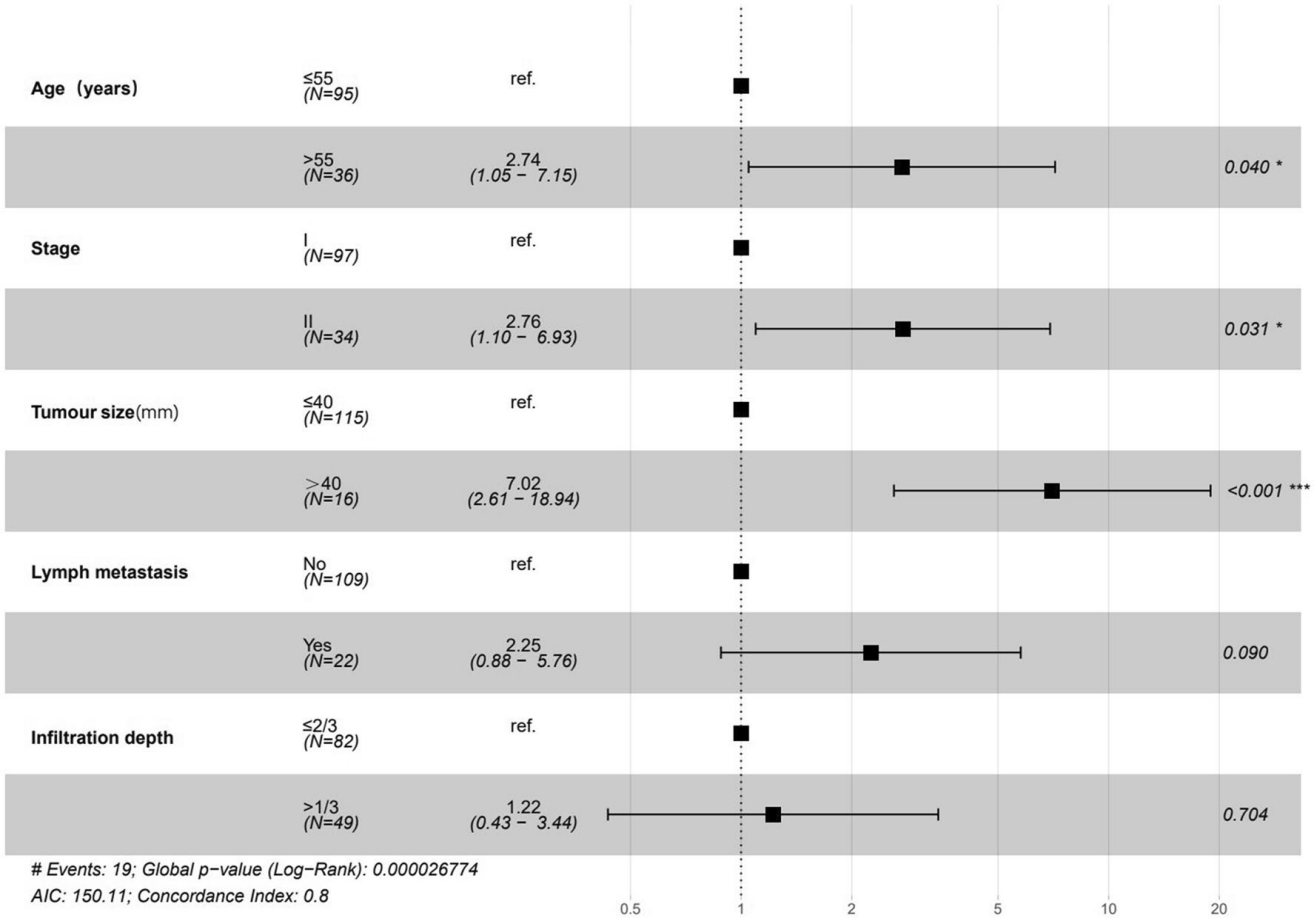
Forest plot shows the multivariate Cox regression model that predicts recurrence of CA

Of all 131 patients, the median follow-up was 43 months, and disease recurrence occurred in 19(14.5%) patients. We firstly analyzed the tumor characteristics associated with RFS. In univariable analysis, age > 55, FIGO stage II, tumor size > 40 mm, positive lymph metastasis, number of positive LNs, depth of invasion > 2/3, positive resection margin and with radiation were risk factors for RFS (*p* < 0.05) (Table 3). In multivariable analysis, age of > 55 (HR 2.74, 95% CI 1.05–7.15, *p* = 0.040), stage II (HR 2.76, 95% CI 1.1–6.93, *p* = 0.031), larger tumor size (HR 7.02, 95% CI 2.61–18.94, *p* < 0.001) were identified as independent predictors of RFS in CA patients. Depth of invasion (HR 1.22, 95% CI 0.43–3.44, *p* = 0.704) and lymph metastasis (HR 2.25, 95% CI 0.88–5.76, *p* = 0.090) was not identified as advantageous factor for prognosis. The results are shown in the forest plot (Fig. 1).

**Table 3:** Univariable Cox regression of recurrence model

Characteristics	HR	(95% CI for HR)	*p* value
Age (years)			
≤ 55	Reference		
> 55	2.48	1.01–6.1	0.049
Stages			
I	Reference		
II	3.49	1.41–8.6	0.007
Surgery manner			
Laparotomy	Reference		
Laparoscopy	2.43	0.68–8.69	0.173
Tumor size (mm)			
≤ 40	Reference		
> 40	6.02	2.36–15.32	< 0.001
Differentiation			
Well differentiated	Reference		
Moderate differentiated	1.61	0.35–7.45	0.545
Poorly differentiated	2.37	0.50–11.17	0.276
Lymph metastasis			
No	Reference		
Yes	3.93	1.59–9.7	0.003
Number of positive LNs	1.46	1.28–1.68	< 0.001
Vascular invasion			
No	Reference		
Yes	1.78	0.64–4.96	0.268
Infiltration depth			
≤ 2/3	Reference		
> 2/3	2.95	1.11–7.81	0.03
Resection margin			
Negative	Reference		
Positive	4.83	1.11–20.98	0.036
Radiation			
No	Reference		
Yes	2.73	1.09–6.8	0.032
Chemotherapy			
No	Reference		
Yes	1.29	0.53–3.19	0.576
d-Dimer (mg/L)	0.99	0.92–1.06	0.729
Platelet (10^9^/L)	1	0.99–1	0.379
TC (mmol/L)	0.89	0.64–1.25	0.515
TAG (mmol/L)	0.93	0.66–1.3	0.671
HDL (mmol/L)	1.35	0.43–4.21	0.61
LDL (mmol/L)	0.69	0.42–1.12	0.135
GLU (mmol/L)	0.99	0.92–1.06	0.707
HB (g/L)	1.01	0.98–1.04	0.437
HB after (g/L)	1	1–1.01	0.89
RBC (10^12^/L)	0.83	0.33–2.1	0.693
RBC after (10^12^/L)	1.76	0.77–4.01	0.181

### Nomograms and internal validation of recurrence model

**Fig. 2:**
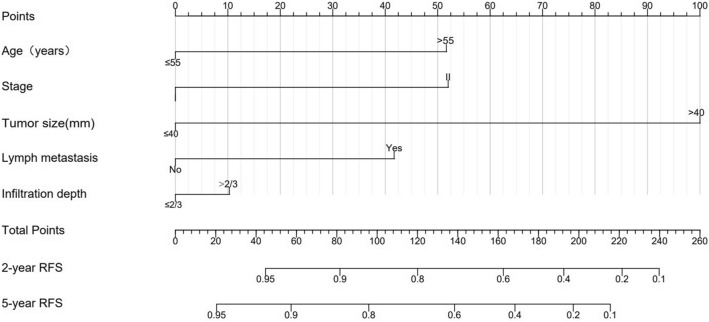
Nomogram for predicting the 2- and 5-year probability of RFS. Draw a vertical line from each variable to the corresponding points scale to obtain its points. The points are then summed and a line is drawn downward from the total points line to obtain the probability of 2- and 5-year RFS

Subsequently, we construct models based on the independent factors screened above. Due to the strong correlation between lymph metastasis and the number of positive LNs, only one variable was chosen in the development of the model. To refine the clinical application of the model, we compared the predictive effect of the models with different variables (Table S1). Finally, the model with the highest C-index was selected. The C-index for the nomogram as the final model is 0.818 (95% CI 0.708–0.928). Thus, five variables including age, FIGO stage, tumor size, lymph metastasis, and invasion depth were used to construct the static nomograms and web-based dynamic nomograms of the recurrence model. The probability of 2- and 5-year RFS was shown in the nomogram (Fig. 2). We conducted sensitivity analysis on the complete data of the recurrence model, and the model achieved similar discrimination. C-index is 0.85 (95% CI 0.73–0.96) (Table S2).

**Fig. 3:**
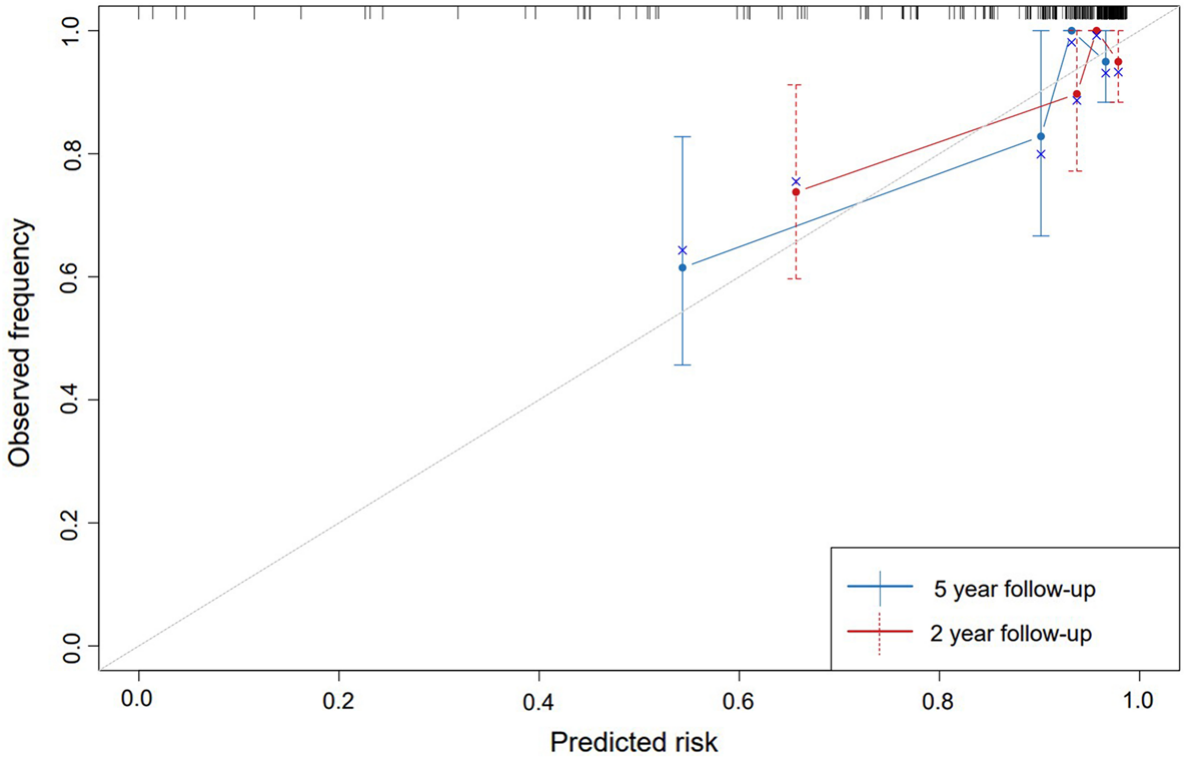
Calibration Curve for the 2, 5 Year recurrence rate from Nomogram. The gray line represents the ideal fit. The nomogram predicted probability of recurrence is plotted on the *x*-axis, and the actual recurrence rate is plotted on the *y*-axis. The dashed and solid line represents the performance of the present nomogram of 2 year and 5 year, respectively. The closer the distance between the two lines, the higher the prediction accuracy

To verify the accuracy of the model, internal verification was performed and calibration curves were drawn. The optimism-adjusted c-statistics for 2 and 5 years were 75.41% and 74.49% after internal validations by bootstrap resampling, and the calibration curve showed good agreement between predictions and observation of the nomogram, as shown in Fig. 3, which indicated that the predictive model has sufficient discriminatory power.

### Cox regression analysis of survival

**Fig. 4:**
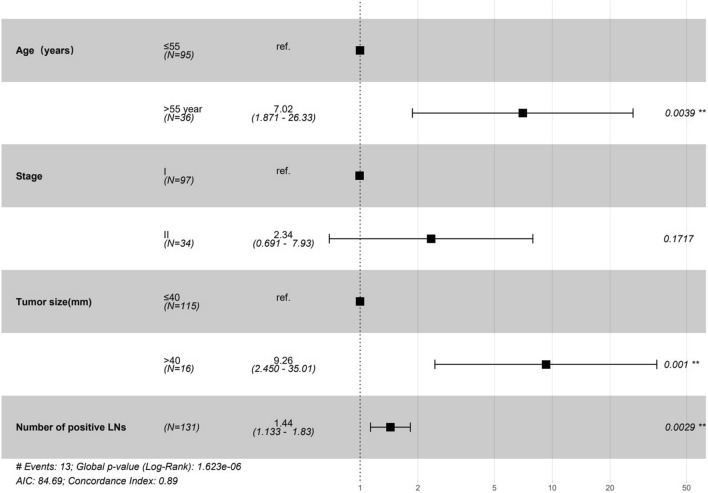
Forest plot shows the multivariate Cox regression model that predicts CSD in the survival model. *CSD* cancer-specific death

Among all patients, the median follow-up was 43 months, and 13 (9.9%) patients suffered death. Initially, we analyzed the tumor characteristics associated with CSS. In univariable analysis, age > 55, FIGO stage II, tumor size > 40 mm, positive lymph metastasis and the number of positive LNs, were risk factors for RFS (*p* < 0.05) (Table 4). In multivariable analysis, age of > 55 (HR 7.02, 95% CI 1.87–26.33, *p* = 0.004), stage II (HR 2.34, 95% CI 0.69–7.93, *p* = 0.172), larger tumor size (HR 9.26, 95% CI 2.450–35.01, *p* = 0.001) and the number of positive LNs (HR 1.44, 95% CI 1.133–1.83, *p* = 0.003) was associated with poor prognosis. The results are shown in the forest plot (Fig. 4).

**Table 4:** Univariable Cox regression of survival model

Characteristics	HR	(95% CI for HR)	*p* value
Age (years)			
≤ 55	Reference		
> 55	3.38	1.13–10.06	0.029
Stages			
I	Reference		
II	3.46	1.16–10.34	0.026
Surgery manner			
Laparotomy	Reference		
Laparoscopy	1.62	0.19–13.77	0.657
Tumour size (mm)			
≤ 40	Reference		
> 40	6.25	2.04–19.16	0.001
Differentiation			
Well differentiated	Reference		
Moderate differentiated	0.98	0.20–4.93	0.985
Poorly differentiated	1.45	0.28–7.52	0.659
Lymph metastasis			
No	Reference		
Yes	4.41	1.47–13.23	0.008
Number of positive LNs	1.48	1.25–1.76	0
Vascular invasion			
No	Reference		
Yes	1.41	0.39–5.14	0.602
Infiltration depth			
≤ 2/3	Reference		
> 2/3	2.58	0.78–8.5	0.12
Resection margin			
Negative	Reference		
Positive	2.39	0.31–18.47	0.404
Radiation			
No	Reference		
Yes	2.57	0.84–7.92	0.1
Chemotherapy			
No	Reference		
Yes	1.92	0.63–5.89	0.251
d-Dimer (mg/L)	0.97	0.71–1.35	0.873
Platelet (10^9^/L)	1	0.99–1	0.494
TC (mmol/L)	0.8	0.54–1.2	0.289
TAG (mmol/L)	0.83	0.5–1.37	0.466
HDL (mmol/L)	1.27	0.33–4.94	0.727
LDL (mmol/L)	0.7	0.39–1.23	0.215
GLU (mmol/L)	0.99	0.94–1.05	0.773
HB (g/L)	1	0.97–1.03	1
HB after (g/L)	1	0.99–1.01	0.808
RBC (10^12^/L)	0.56	0.19–1.65	0.293
RBC after (10^12^/L)	2.28	0.85–6.14	0.101

### Nomograms and validation of survival model

**Fig. 5:**
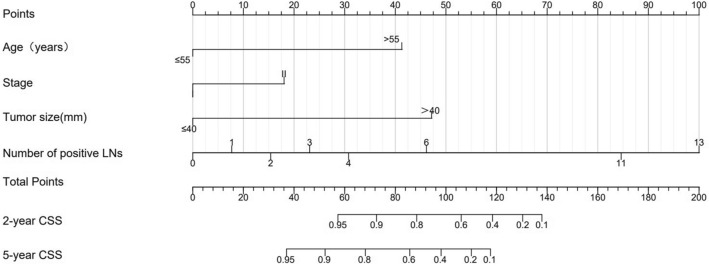
Nomogram for predicting the 2- and 5-year probability of CSS. *CSS* cancer-specific survival

We then develop a survival model nomogram based on the above analysis. To refine the clinical application of the model, the predictive effect of the models with different variables was compared (Table S3). The variable selection of the model was based on the comprehensive consideration of the results of single factor analysis to find the variables that have an impact on the prognosis, the correlation between the variables and the clinical significance. Finally, the model with the highest C-index was selected as the final model, with a C-index of 0.896 (95% CI 0.806–0.986). Four variables including age, FIGO stage, tumor size and the number of positive LNs were used to construct the static nomograms and web-based dynamic nomograms of the survival model. The nomogram shows the probability of 2- and 5-year CSS (Fig. 5). We conducted sensitivity analysis on the complete data of the survival model, and the model achieved similar discrimination. C-index is 0.92 (95% CI 0.81–1.00) (Table S4).

**Fig. 6:**
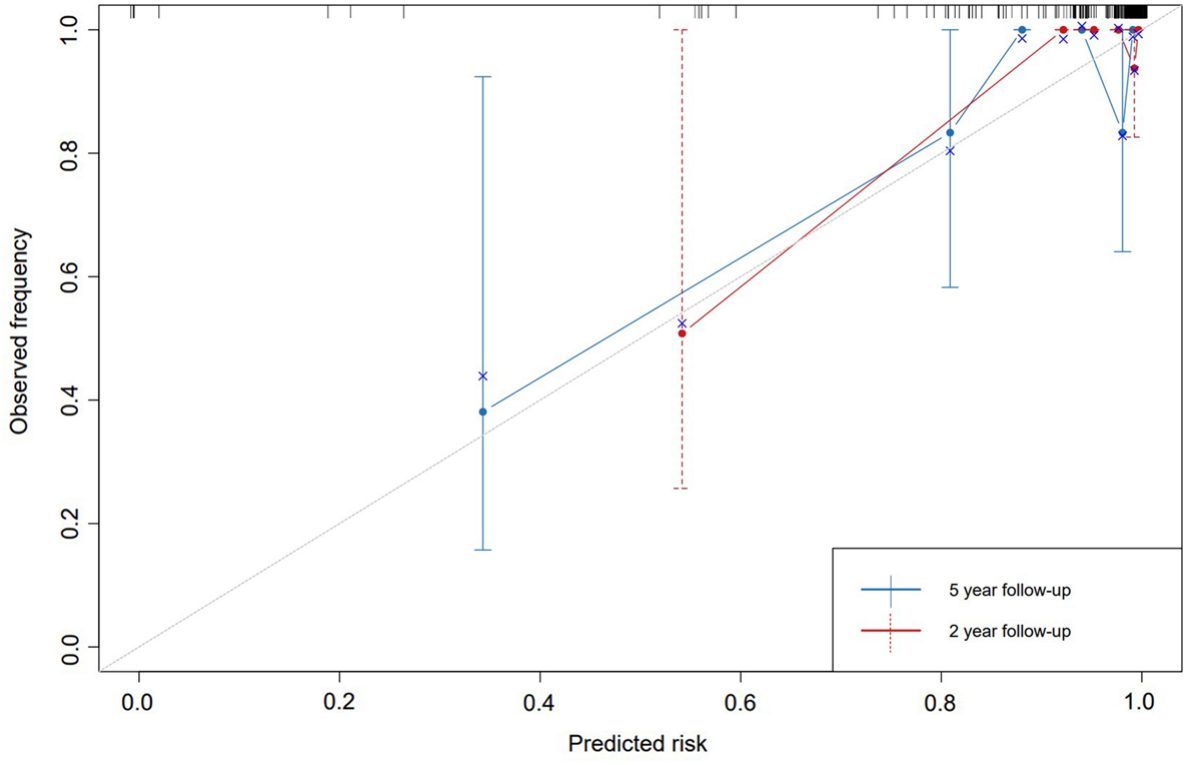
Calibration Curve for the 2, 5 Year CSS from Nomogram. The gray line represents the ideal fit. The nomogram predicted CSS is plotted on the *x*-axis, and the actual CSS is plotted on the *y*-axis. The dashed and solid line represents the performance of the present nomogram of 2 year and 5 year, respectively

We then performed internal validation using the bootstrap resampling method and drew calibration curves (Fig. 6), with optimism-adjusted c-statistics of 83.22% and 83.31% for the 2-year and 5-year CSS, respectively, indicating that the predictive model has sufficient discriminatory power. Additionally, we performed an external validation using SEER database. A total of 1679 patient data from SEER were included in this study and used for external validation. Compared with our data, more patients in the SEER database were no older than 55 years old (80.8%, *n* = 1356), and more patients were in stage I (93.2%, *n* = 1564). There was no significant difference in tumor size between the two cohorts. Clinical characteristics of the SEER cohort and the original data cohort are shown in Table S5. After external validation, the C-index of the nomogram predicting 2- and 5-year CSS was 0.69 and 0.71, respectively.

We could use the two nomograms to predict the RFS and CSS of patients with CA, respectively. For instance, a 50-year-old patient with a primary tumor of 45 mm in size (100 points), stage II (52 points), no lymph metastasis (0 points), and invasion depth ≤ 2/3, had a total of 152 points. Correspondingly, the 2- and 5-year RFS probabilities were 66% and 51%, respectively. We have developed two web-based calculators in order to simplify the application of the model, (https://yfycrc.shinyapps.io/recurrence_rate/; https://yfycrc.shinyapps.io/survival/).

## Discussion

The incidence of cervical adenocarcinoma has increased over the past 2 decades (Siegel et al. 2021). A large body of evidence suggests that the overall prognosis of cervical adenocarcinoma is worse than that of cervical squamous cell carcinoma (Lee et al. 2011; Rose et al. 2014), therefore, the identification of prognostic factors and the development of predictive models are important to optimize treatment planning and guidance of CA patients.

A prognostic nomogram is a predictive model that has been widely used in recent years to estimate the prognosis of cancer (El Sharouni et al. 2021). This novel model has been used to tailor the prognosis of cervical cancer(Wang et al. 2018; Xie et al. 2020; Zhou et al. 2015). Shim’s research constructed a nomogram to predict 5-year OS of patients with cervical cancer with a C-index of 0.69 (Shim et al. 2013). Lee’s study analyzed 1702 patients with stage IB–IIA cervical cancer who underwent adjuvant radiotherapy after radical hysterectomy and constructed a nomogram to predict 5-year OS with a C-index of 0.69 (Lee et al. 2013). However, few studies have focused on CA. Recently, Ni et al. constructed nomograms predicting 2- and 5-year CSS in patients with cervical adenocarcinoma using SEER dates with adjusted C-statistics of 0.90 and 0.89, respectively (Ni et al. 2021). In their study, they used only a public database (SEER) with few variables involved and performed a prediction model for survival only, without predicting recurrence.

In our current study, age, stage, size of the tumor, lymph metastasis and depth of invasion were identified as the recurrence-related factors for CA, which is consistent with the results of other studies (Lee et al. 2017; Levinson et al. 2021; Yoneoka et al. 2021). In the nomogram, tumor size contributed the most to RFS, followed by stage and age. Lymph metastasis and infiltration depth were also established as independent prognostic factors. In a study of SCC by Levinson et al., lymphovascular space invasion, tumor size and depth of invasion were found to be associated with recurrence (Levinson et al. 2021). Among these factors, the depth of invasion had the greatest impact on the prognosis, which is different from our research on CA.

We then explored the prognostic factors associated with CA survival and found that age, FIGO stage, tumor size and number of positive LNs were independent predictors of survival in CA. Histological type, age, FIGO stage, tumor size, stromal invasion, lymphatic-vascular space infiltration (LVSI), parametrial involvement, and concurrent chemotherapy, have been identified and included in the prediction model related to survival in previous cervical cancer studies (Lee et al. 2013; Polterauer et al. 2012; Shim et al. 2013; Zhou et al. 2015). In our study, these four factors: age, stage, size of the tumor, and the number of positive LNs, were identified as independent factors for patient survival and were incorporated into the model, which is consistent with the results of other studies (Gadducci et al. 2019; Khalil et al. 2015; Park et al. 2010; Stolnicu et al. 2019).In the current nomogram, the number of positive LNs contributed the most to prognosis, followed by tumor size and age. The tumor stage was established as another independent prognostic factor, although it is also a related factor to tumor size. Zhou et al. found that in patients with stage I–IIB ECA, tumor diameter (≥ 4 cm) and the number of positive lymph nodes were independent prognostic factors of relapse free survival (RFS), while the positive number of pelvic lymph nodes and age of operation were independent prognostic factors of OS (Zhou et al. 2018).

We have established the survival model through internal verification and external verification. Since the SEER database does not record the recurrence of patients, it cannot be used for external verification of our recurrence model. Both models exhibited satisfactory performance with accurate discrimination. In these models, each prognostic factor is quantified and visualized by static nomograms that can individually predict 2-year and 5-year RFS and CSS in CA patients. Two web-based calculators were developed (https://yfycrc.shinyapps.io/recurrence_rate/; https://yfycrc.shinyapps.io/survival/). After entering the appropriate variables, the patient's RFS or CCS and 95% CI can be obtained. Based on these two predictive models, physicians can determine individual risk, predict outcomes, and select appropriate therapies for patients with CA.

There are some limitations in the study. First, we established this model through retrospective analysis, which may lead to bias due to the lack of random assignment, and some missing values. Second, because all patients were from an East Asian population, the corresponding ethnic susceptibility is unknown; our results should be extrapolated to other populations with caution. Third, the prediction model for tumor recurrence was internally validated, so additional external validation using cohorts from different hospitals or regions is needed. Fourth, Due to the limited data, we did not divide the data into training set and test set, considering that the modeling data would be reduced after dismantling and the degree of assurance of model modeling and verification would be reduced. In the future, on the basis of increasing the sample size, more adequate internal verification can be carried out.

In conclusion, in the current study, we developed and validated nomogram models to predict 2-year and 5-year RFS and CSS in patients with early-stage CA, respectively. This will help to assess the prognosis of patients with CA more accurately evaluate in clinical work.

## Supplementary Information

Below is the link to the electronic supplementary material.

Supplementary file1 (XLSX 14 KB)

Supplementary file2 (XLSX 10 KB)

Supplementary file3 (XLSX 12 KB)

Supplementary file4 (XLSX 10 KB)

Supplementary file5 (XLSX 11 KB)

## Data Availability

The data from the current study are available from the corresponding author on reasonable request.
